# Supplementary material for: Rapid and tunable method to temporally control gene editing based on conditional Cas9 stabilization
Source: Nat Commun. 2017 Feb 22;8:14370. doi: 10.1038/ncomms14370 (PMC5322564; doi:10.1038/ncomms14370)
Supplement: Supplementary Information — Supplementary Figures and Supplementary Tables [file ncomms14370-s1.pdf]

Supplementary Figure 1

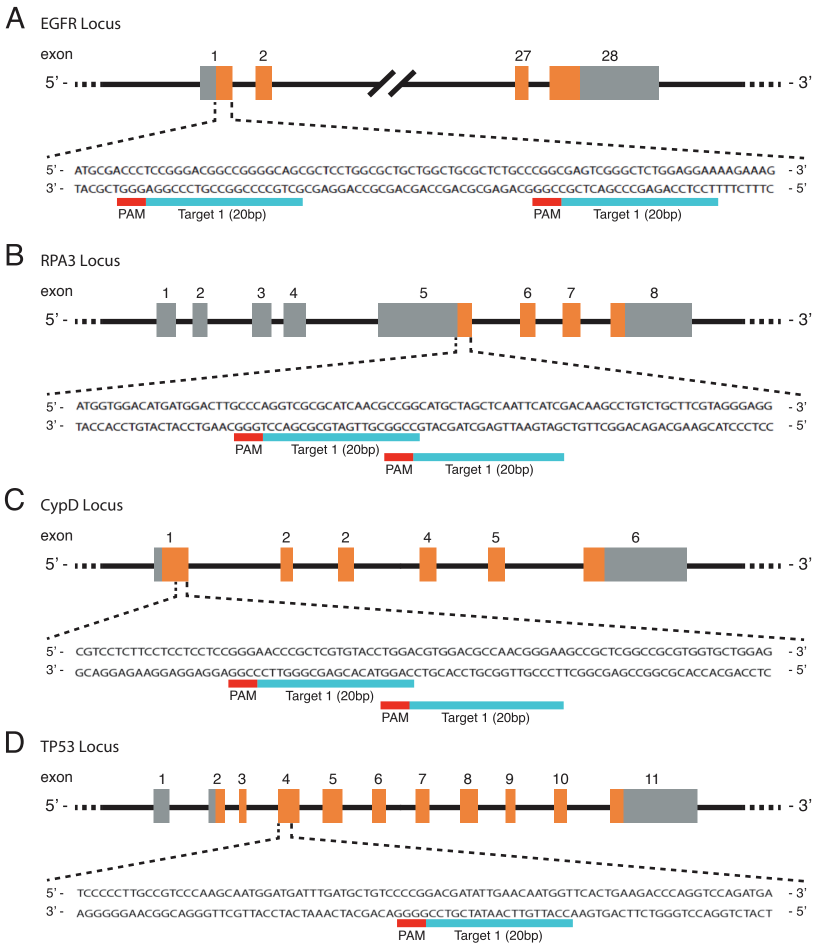

Supplementary Figure 1. Schematic of location of small guide RNAs on EGFR, RPA3, CYPD and TP53 genes loci. Guide RNA predictions were performed using Optimized CRISPR design tool from MIT (http://crispr.mit.edu)

Supplementary Figure 2

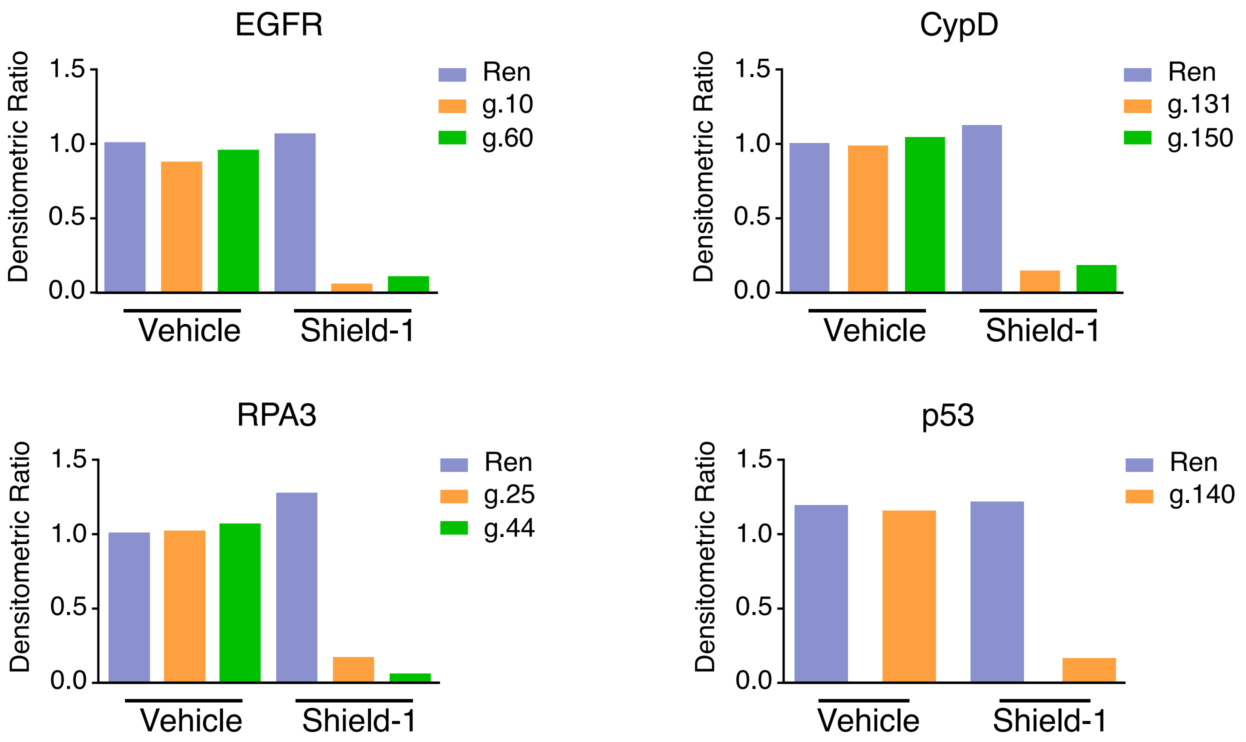

Supplementary Figure 2. Quantification of protein levels by LICOR Odyssey imaging. Infrared fluroscent signals which are directly proprotional to the amount of antigen on western blots were quantified using ratiometric detection option of Odyssey V3.0 software. Individual proetin levels were normalized to the loading control α-tubulin.

Supplementary Figure 3

A

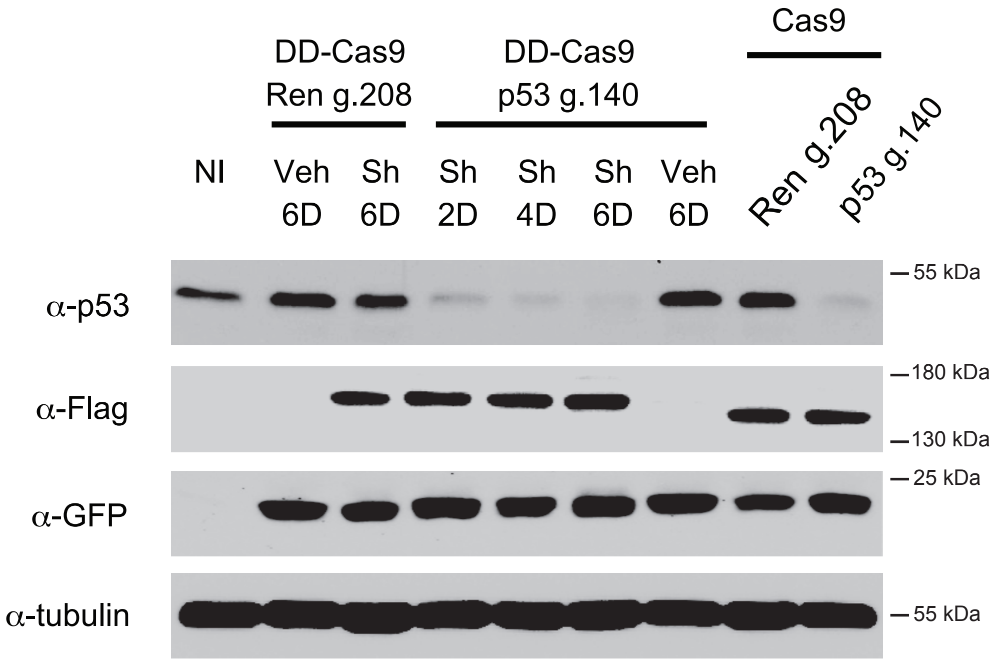

B

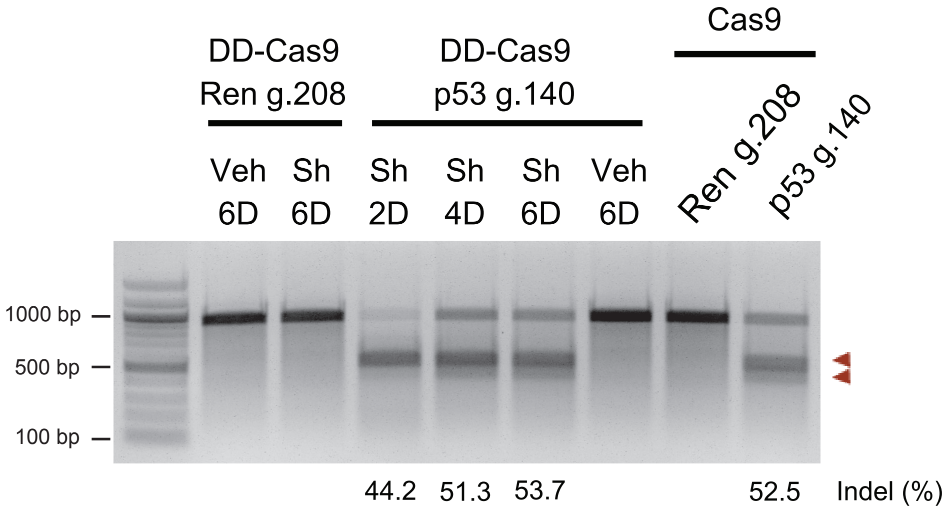

Supplementary Figure 3. Comparison of destabilzed-Cas9 (DD-Cas9) versus constitutive-Cas9 (Cas9) activity. **A.** Western blot analysis of A549 lysates infected either with destabilized-Cas9 or constitutive-Cas9. Cells infected with destabilized-Cas9 lentiviruses targeting either Ren as a control or p53 gene were treated with Shield-1 (Sh) or Vehicle (Veh) and lysates were collected at days (D) as indicated, knock-down was measured by western blot. Similarly, constitutive-Cas9 expressing lentivirus particles were used to infect A549 cells and knock-down efficiency was analyzed by western blot after 6 days. Similar infection rates were aimed and achieved for each virus as quantified by FACS (not shown). Results indicate similar activity destabilized-Cas9 and constitutive-Cas9. NI: not infected. **B.** Surveyor assay on the same samples as in (A) shows similar genome editing measured by indel formation.

Supplementary Figure 4

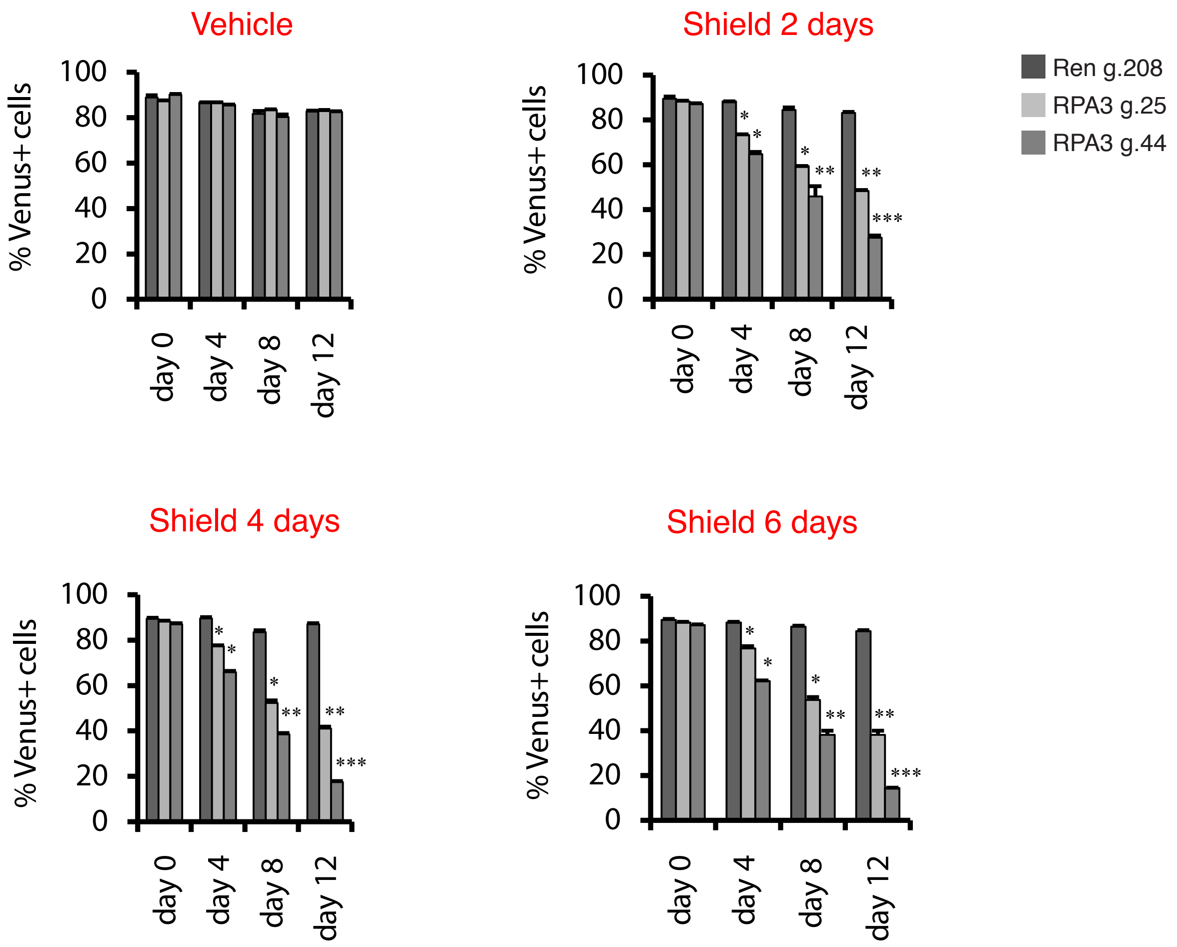

Supplementary Figure 4. DD-Cas9 can be coupled to a modified fluroscent protein to generate a traceable system. A549 cells were transduced with DD-Cas9/P2A/Venus lentiviral vector. Cells were then treated with Shield-1 or vehicle control and quantified by FACS. The panels illustrate the percentage of Venus^{+} cells during time in A549 cells transduced with the DD-Cas9/P2A/Venus lentiviral vector targeting RPA3 gene with two individual sgRNAs. Upon treatment with Shield-1 and vehicle control for different time points, it is evident that only 2 days of Shield-1 treatment was sufficient to induce similar changes with 4 and 6 days continuous ligand treatment.Error bars are standard deviation, (n=3 for each condition, p values: *$p<0.05$, **$p<0.01$, ***$p<0.005$) Student’s t-test.

Supplementary Figure 5

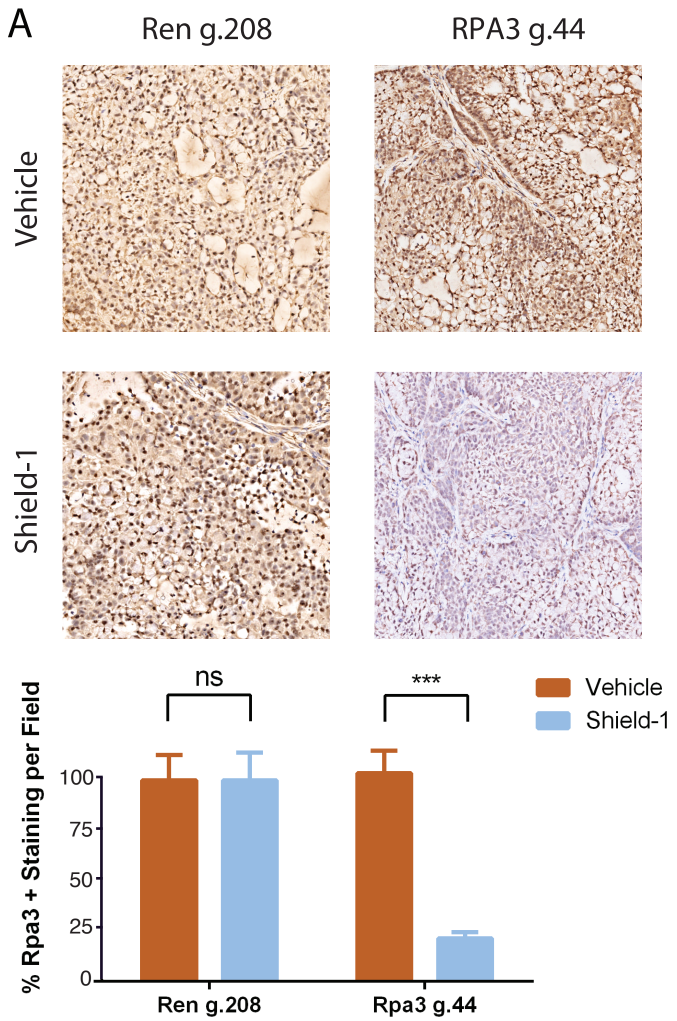

Supplementary Figure 5. Targeting RPA3 in A549 cell line xenografts resulted in dramatic decrease in the RPA3 staining following the treatment with the Shield-1. A549 cells were transduced with the DD-Cas9 vector targeting the RPA3 locus and as control the Renilla gene and transplanted sub-cutaneously in immune-deficient mice. Tumors were extratced 10 days after initial Shield-1 treatment and processed for IHC. Tumor staining are derived from the same samples shown in Figure 3F. Quantification of 4 different fields on the stained tumor section in presented on the lower panels. Error bars are standard deviation, (n=4, ***p<0.005) Student's t-test.

Supplementary Figure 6

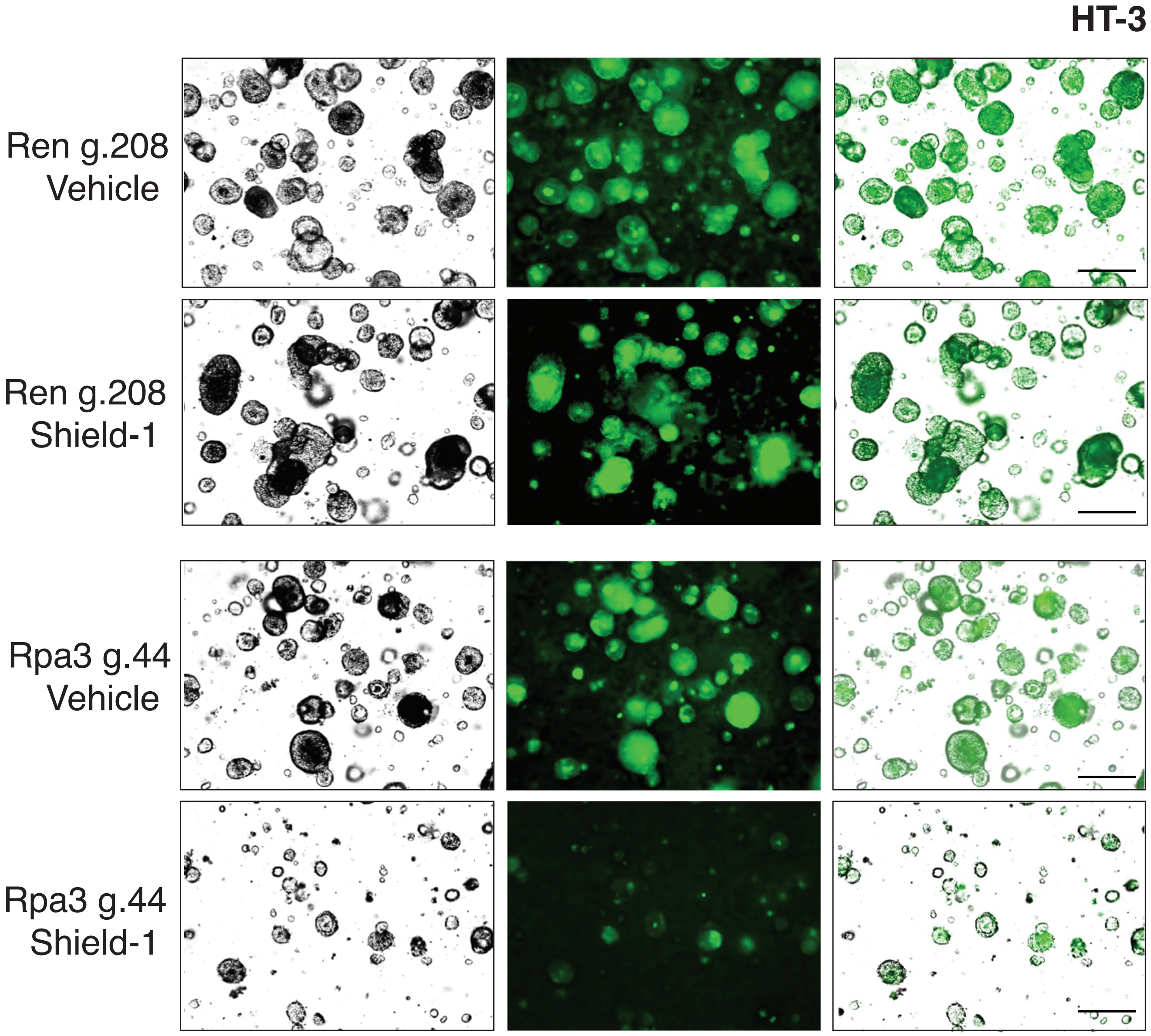

Supplementary Figure 6. DD-Cas9/P2A/Venus could be used to infect primary human tumor derived cultures and to assess their cellular vulnerabilities. Human pancreatic cancer derived organoids were infected with the DD-Cas9/P2A/Venus lentiviral vector targeting RPA3 and as control Renilla gene. Organoids, cultured in 24-well plates, were treated with Shield-1 or vehicle control for 72 hours post-infection. Number of Venus^{+} organoids was determined following 6 days of Shield-1 treatment. The picture depict representative images of the organoids. Scale bars, 500 μm.

Supplementary Figure 7

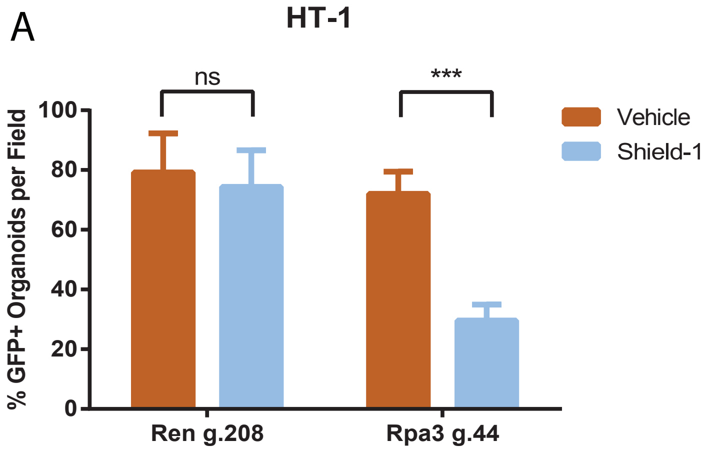

Supplementary Figure 7. Quantification of human pancreatic cancer derived organoids survival after RPA3 knock-out **(A)** HT-1 (figure 4F) and **(B)** HT-3 (Supplementary figure 6). HT-1 and HT-3 were infected with the DD-Cas9/P2A/Venus lentiviral vector targeting RPA3 and as control Renilla gene. Organoids, cultured in 24-well plates, were treated with Shield-1 or vehicle control for 72 hours post-infection. Number of Venus$^+$ organoids was determined following 6 days of Shield-1 treatment. Quantification of 5 different fields. Error bars are standard deviation, (n=5, ***p<0.005) Student's t-test.

Supplementary Figure 8

ct_all	ct_1	ct_2	ct_3	ct_4	ct_5	ct_6	ct_7	ct_8	alignment		
275841	41912	38007	26877	12993	25942	48982	61961	19167	< no length change >		
94828	22	13	36685	24116	16358	104	20	17510	CAATGGATGATTTGATGCTGTCCCCGGA	A	ACGATATTGAACAATGGTTCACTGAAGAC
24663	5	5	9942	6169	4523	14	7	3998	CAATGGATGATTTGATGCTGTCCCCG		ATTGAACAATGGTTCACTGAAGAC
19084	7	3	8177	4434	3036	18	1	3408	CAATGGATGATTTGATGCTGTCCCCGGA	A	ACGATATTGAACAATGGTTCACTGAAAAC
7796	2	2	3215	1917	1312	5	3	1340	CAATGGATGATTTGATGCTGTCCCCGG		ATTGAACAATGGTTCACTGAAGAC
5541	2	1	2304	1416	944	2	0	872	CAATGGATGATTTGATGCTGTCCCCGGA		ATTGAACAATGGTTCACTGAAGAC
4115	1	1	1827	953	595	4	1	733	CAATGGATGATTTGATGCTGTCCCCGGA	A	ACGATATTGAACAATGGTTCACTGAACAC
3673	0	0	1489	987	614	3	2	578	CAATGGATGATTTGATGCTGTCCCCGGA	[62bp]	
2811	1	0	1221	659	427	3	0	500	CAATGGATGATTTGATGCTGTCCCCGGA	A	ACGATATTGAACAATGGTTCACTGAATAC
2401	1	0	942	467	502	3	2	484	CAATGGATGATTTGATGCTGTCCCC		
2311	1	0	948	675	360	1	1	325	CAATGGATGATTTGATGCTGTCCCCG	[60bp]	
1945	1	2	717	502	326	1	0	396	CAATGGATGATTTGATGCTGTCCCCGGA	[60bp]	
1848	0	0	740	501	294	0	1	312	CAATGGATGATTTGATGCTGTCCCCGG		
1628	0	0	667	423	257	1	0	280	CAATGGATGATTTGATGCTGTCCCCGG		GATATTGAACAATGGTTCACTGAAGAC
1589	0	0	556	514	233	2	0	284	CAATGGATGATTTGATG		
1559	0	1	551	425	285	1	0	296	CAATGGATGATTTGATGCTGTCCCCG		ACGATATTGAACAATGGTTCACTGAAGAC
1456	0	0	587	409	223	0	1	236	CAATGGATGATTTGATGCTGTCCCCGGA	AA	AACGATATTGAACAATGGTTCACTGAAGAC
1405	0	0	591	402	207	4	0	201	CAATGGATGATTTGATGCTGTCCCCGGA	[63bp]	
1380	0	0	523	329	256	1	0	271	CAATGGATGATTTGAT		TCACTGAAGAC
1318	0	0	534	293	219	0	0	272	CAATGGATGATTTGATGCTGTCCCC		GATATTGAACAATGGTTCACTGAAGAC
1303	1	0	509	318	212	1	0	262	CAATGGATGATTTGATGCTGTCCCCGGA	A	ACGATATTGAACAATGGTTCACTTAAGAC

Supplementary Figure 8. An alignment of the 20 most frequent observed patterns near the Cas9 target sequence, as well as counts of these patterns for each of the eight samples are shown.

Supplementary Figure 9

Figure 1C

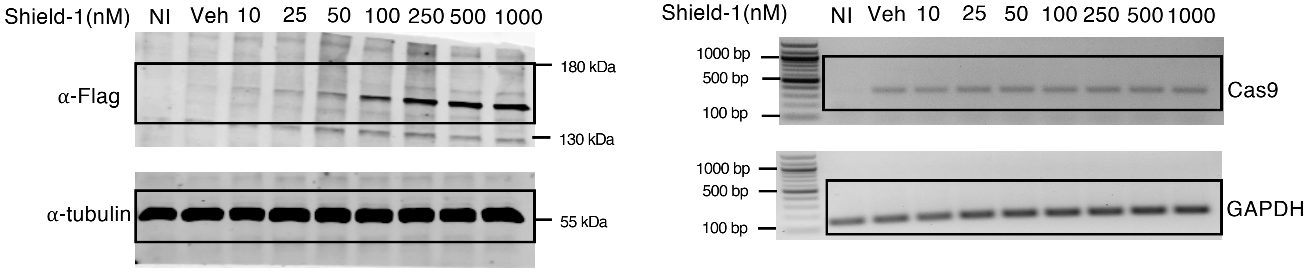

Figure 1D

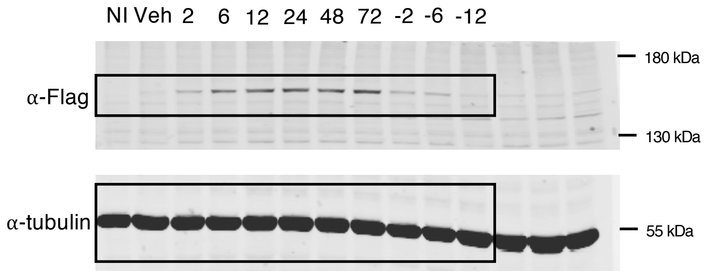

Figure 2A

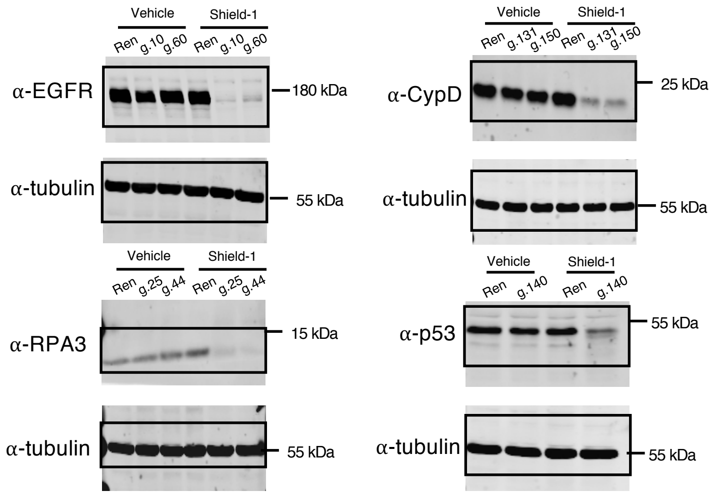

Figure 2B

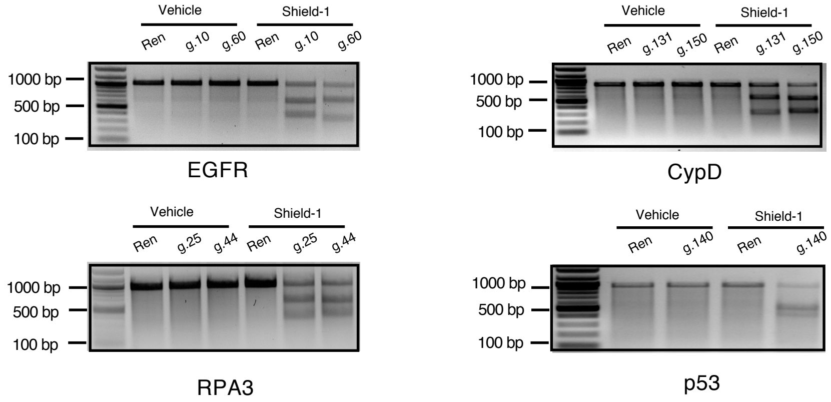

Figure 4B

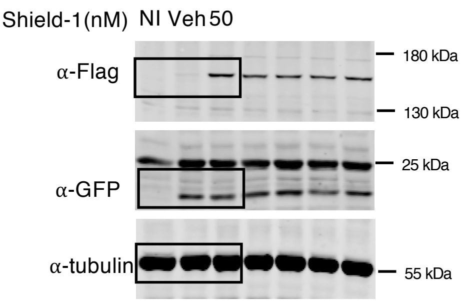

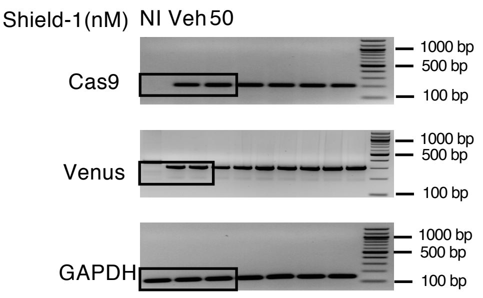

Supplementary Figure 3A

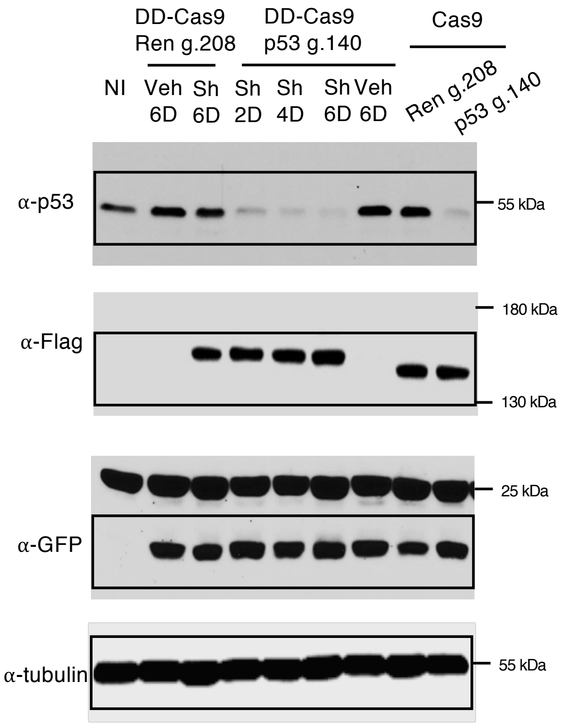

Supplementary Figure 3B

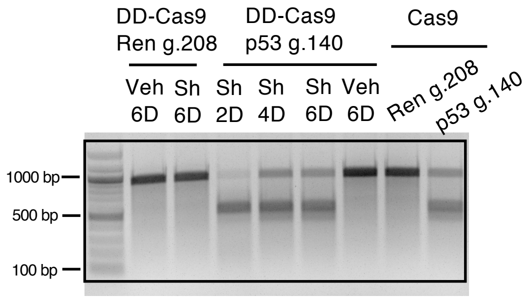

Supplementary Figure 9. Uncropped images of all the western blots and agarose gels used in this study.

Supplementary Table 1. Complementary oligonucleotides used for cloning sgRNAs

Oligo	Sequence
CypD g.131 F	5'- CACCgCAGGTACACGAGCGGGTTCC -3'
CypD g.131 R	5'- AAACGGAACCCGCTCGTGTACCTGc -3'
CypD g.150 F	5'- CACCgTCCCGTTGGCGTCCACGTCC -3'
CypD g.150 R	5'- AAACGGACGTGGACGCCAACGGGAc -3'
EGFR g.10 F	5'- CACCgGCTGCCCCGGCCGTCCCGGA -3'
EGFR g.10 R	5'- AAACTCCGGGACGGCCGGGGCAGCc -3'
EGFR g.60 F	5'- CACCgTCCTCCAGAGCCCGACTCGC -3'
EGFR g.60 R	5'- AAACGCGAGTCGGGCTCTGGAGGAc -3'
RPA3 g.25 F	5'- CACCgCCGGCGTTGATGCGCGACCT -3'
RPA3 g.25 R	5'- AAACAGGTCGCGCATCAACGCCGGc -3'
RPA3 g.44 F	5'- CACCgGATGAATTGAGCTAGCATGC -3'
RPA3 g.44 R	5'- AAACGCATGCTAGCTCAATTCATCc -3'
TP53 g.140 F	5'- CACCgCCATTGTTCAATATCGTCCG -3'
TP53 g.140 R	5'- AAACCGGACGATATTGAACAATGGc -3'
tdTOM g.230 F	5'- CACCgTTGTAATCGGGGATGTCGGC -3'
tdTOM g.230 R	5'- AAACGCCGACATCCCCGATTACAAc -3'
tdTOM g.282 F	5'- CACCgGGAGCGCGTGATGAACTTCG -3'
tdTOM g.282 R	5'- AAACCGAAGTTCATCACGCGCTCCc -3'
tdTOM g.292 F	5'- CACCgGAACTTCGAGGACGGCGGTC -3'
tdTOM g.292 R	5'- AAACGACCGCCGTCCTCGAAGTTCc -3'

Supplementary Table 2. RT-PCR primer sets

Oligo	Sequence
SpCas9-F1	5'- TGGAAGAGTCCTTCCTGGTG -3'
SpCas9-R1	5'- CGAACAGGCCATTCTTCTTC -3'
SpCas9-F2	5'- CCCAAGAGGAACAGCGATAA -3'
SpCas9-R2	5'- TTGGCTTCCAGAAAGTCGAT -3'
Venus-F	5'- AAGCTGACCCTGAAGCTGATCTGC -3'
Venus-R	5'- CTTGTAGTTGCCGTCGTCCTTGAA -3'
GAPDH-F	5'- GGCTGAGAACGGGAAGCTTGTCAT -3'
GAPDH-R	5'- CAGCCTTCTCCATGGTGGTGAAGA -3'

Supplementary Table 3. PCR primers for surveyor assay

Oligo	Sequence
EGFR-SVR-F	5'- TTGGCTCGACCTGGACATAG -3'
EGFR-SVR-R	5'- GGGGAAAGTGAGGGAAGAAA -3'
RPA3-SVR-F	5'- ATCCTGTGATCGCAGAAAGG -3'
RPA3-SVR-R	5'- CAAGACTTTGGGCCTGTTTC -3'
CYPD-SVR-F	5'- AGGGGTAGTCCACGGACAG -3'
CYPD-SVR-R	5'- CCGCACTACTGCTGGAAAC -3'
TP53-SVR-F	5'- GGGTTGGAAGTGTCTCATGC -3'
TP53-SVR-R	5'- TCAAAAGCCAAGGAATACACG -3'
